# VPg of murine norovirus binds translation initiation factors in infected cells

**DOI:** 10.1186/1743-422X-3-33

**Published:** 2006-05-23

**Authors:** Katie F Daughenbaugh, Christiane E Wobus, Michele E Hardy

**Affiliations:** 1 Veterinary Molecular Biology, Montana State University, Bozeman, MT, USA; 2 Department of Pathology and Immunology, Washington University School of Medicine, St Louis, MO, USA

## Abstract

**Background:**

Norovirus genomic and subgenomic RNAs are covalently linked at the 5' nucleotide to a 15 kD protein called VPg. VPg of two human norovirus strains binds translation initiation factor eIF3 and other eIFs *in vitro*, suggesting VPg functions in initiation of protein synthesis on viral RNA. Human norovirus strains are not cultivable, and thus experimental evidence of interactions between VPg and eIFs in infected cells has been lacking. We used the cultivable murine norovirus MNV-1 as a model to study interactions between VPg and eIFs in infected cells.

**Results:**

As shown previously for human norovirus VPg, MNV-1 VPg bound eIF3, eIF4GI, eIF4E, and S6 ribosomal protein in cell extracts by GST pull-down assay. Importantly, MNV-1 VPg co-precipitated eIF4GI and eIF4E from infected macrophages, providing evidence that VPg interacts with components of the translation machinery in norovirus infected cells.

**Conclusion:**

The interactions between MNV-1 VPg and eIFs completely mimic those reported for the human norovirus VPg, illustrating the utility of MNV-1 as a relevant molecular model to study mechanisms of human norovirus replication.

## Background

*Noroviruses *constitute a genus in the family *Caliciviridae *and are the most frequent cause of foodborne viral gastroenteritis epidemics [1]. The norovirus genome is a 7.7 kb, positive-sense, single-stranded RNA that is polyadenylated at the 3' end. The genome codes for three open reading frames. ORF1 encodes the nonstructural proteins that are synthesized as a polyprotein precursor and processed by the 3C-like viral protease. ORF2 and ORF3 encode the capsid protein VP1 and minor structural protein VP2, respectively. Both VP1 and VP2 are synthesized from a subgenomic RNA that is 3' coterminal with the genomic RNA. Instead of a 7-methylguanosine (m^7^G) cap structure at the 5' end, genomic and subgenomic RNAs are covalently linked to a viral protein called VPg, for viral protein genome-linked. The linkage between VPg and the 5' nucleotide has been demonstrated experimentally only for the animal caliciviruses [2,3], but by analogy, it is assumed that VPg also is linked to norovirus genomes. The function(s) of VPg in the norovirus replication cycle are not known, but data that this small protein functions in translation initiation are accumulating. Studies of animal caliciviruses showed VPg is necessary for infectivity of native viral RNA [2], but an m^7^G cap can confer infectivity to *in vitro *synthesized feline calicivirus (FCV) genomic RNA transcripts [4]. Together, data from these studies conclude that the m^7^G cap functionally substitutes for VPg when transfecting calicivirus RNA into cells. We previously reported that VPg of the Norwalk (NV) and Snow Mountain (SMV) strains of human norovirus bound translation initiation factor eIF3, providing the first direct evidence that VPg may function in ribosome recruitment to viral RNA [5]. Recently, an interaction between FCV VPg and translation initiation factor eIF4E was reported, and this interaction was required for translation of FCV VPg-linked RNA in vitro [6].

Most cellular mRNAs are translated by a cap-dependent mechanism driven by protein-protein and protein-RNA interactions between translation initiation factors (eIFs) and mRNA [7]. Cap-dependent translation initiates with binding of the eIF4F complex that consists of eIF4E, eIF4GI and eIF4A, to the m^7^G cap structure. Recruitment of the 43S pre-initiation complex composed of eIF3, 40S ribosomal subunits, and initiator tRNA to mRNA occurs primarily through interactions between eIF4GI and eIF3 [8,9]. This 48S complex scans the mRNA to initiate translation at the first strong context AUG. Cap-independent translation is mediated by extensively structured regions of RNA called internal ribosome entry sites (IRES). These elements are found in genomes of picornaviruses, pestiviruses, and insect viruses of the *Dicistroviridae *family [10], and some cellular mRNAs as well (reviewed in [11]. Initiation complexes assemble internal to the 5' end of the RNA molecule and translation initiates at, or shortly downstream, of the IRES. The fact that norovirus RNAs lack a m^7^G cap, are 5' nucleotide-protein linked, and have 5' untranslated regions (UTR) of only 10 nucleotides suggest translation initiation on norovirus RNA mediated by VPg proceeds in a manner distinct from cap-dependent and IRES-dependent mechanisms.

VPg of human noroviruses binds directly to eIF3, is present in complexes with other eIFs, and inhibits translation of mRNAs that have different eIF requirements for functional initiation complex assembly [5]. All of these data were obtained using purified protein and cell-free assays. Human noroviruses do not grow in cell culture, and therefore, connecting this eIF binding data with events that occur in norovirus infected cells is challenging. A significant advance in the field was the discovery of a murine norovirus (MNV-1) that replicates in cultured macrophages and dendritic cells [12,13]. MNV-1 is the only norovirus that grows in culture, and is closely related genetically to the human norovirus strains [12]. Therefore, we used MNV-1 to address questions about the functional significance of interactions between VPg and eIFs observed with the human noroviruses. In addition, determining whether protein-protein interactions are the same between the human and murine viruses would establish whether MNV-1 constitutes a relevant molecular model for the non-cultivable human strains. Our data show that MNV-1 VPg binds eIFs in infected cells, and that interactions between MNV-1 VPg and eIFs are the same as those that occur between eIFs and human norovirus VPg. These findings indicate that MNV-1 represents an important molecular tool for the study of human noroviruses in tissue culture.

## Results

### Recombinant MNV-1 VPg binds eIF3, eIF4GI, eIF4E, and S6 ribosomal protein in vitro

**Figure 1:**
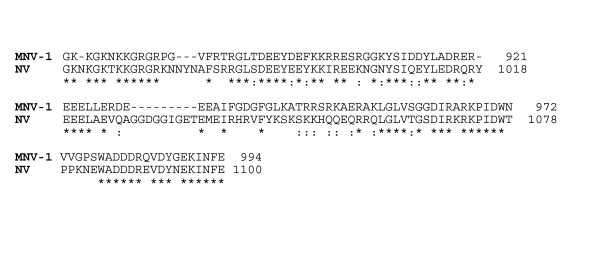
**MNV-1 VPg and NV VPg share significant amino acid sequence identity**. Sequences encoding MNV-1 VPg and NV VPg were aligned with ClustalW. Asterisks indicate identical residues. Semicolons are conservative substitutions. Amino acid numbers correspond to the position in the ORF1 polyprotein.

VPg of genogroup I NV and genogroup II SMV share 68% amino acid sequence identity and both bind purified eIF3 and to eIF3 in cell extracts [5]. VPg sequences of MNV-1 and NV were aligned and share 54% identity (Figure 1). The N- and C-terminal domains showed the highest level of conservation, and flank a central variable region that includes a glycine-rich, nine amino acid insertion in NV VPg. Interpreted in the context of the inferred function of VPg in translation, an overall amino acid sequence identity of 54%, and conservation in both N and C-terminal domains, suggested that MNV-1 could have similar eIF binding properties as NV VPg.

**Figure 2:**
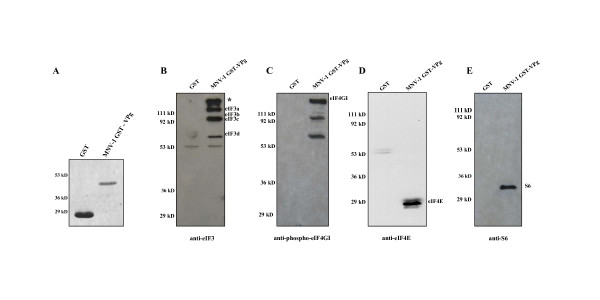
**MNV-1 GST-VPg binds eIF3, phospho-eIF4GI, eIF4E, and S6 ribosomal protein by pull-down assay**. GST and MNV-1 GST-VPg were expressed in bacteria and purified as described in the text. A) Purified GST and GST-VPg, analyzed by 10% SDS-PAGE and stained with Coomassie blue. Proteins in the pull-down eluates were subjected to SDS-PAGE, and then immunoblot probed with (B) anti-eIF3, (C) anti-phospho-eIF4GI, (D) anti-eIF4E, and (E) anti-S6 ribosomal protein. Blots were developed with ECL chemiluminescent substrate. The asterisk in B indicates a protein identified as eIF4GI.

To test if MNV-1 VPg binds eIFs in a cell-free system, VPg was expressed as a GST fusion protein in bacteria, purified (Figure 2A), and used in pull-down assays with extracts prepared from RAW 264.7 macrophages as previously described [5]. Proteins in pull-down eluates were analyzed by immunoblots probed with antibodies to eIF3 (Figure 2B), phospho-eIF4GI (Figure 2C), eIF4E (Figure 2D), and S6 ribosomal protein (Figure 2E). Similar to data obtained for NV VPg, all of these factors bound MNV-1 GST-VPg.

eIF3 is a large complex composed of 12 subunits [7]. Four of these subunits, eIF3a, eIF3b/eIF3c doublet [14], and eIF3d, were identified in the VPg pull-down eluates. Identification was made by the banding pattern that is observed for eIF3 when this polyclonal antibody is used as a probe [14]. The reasons for detecting only a few of the subunits in the eluates are not known, but this large complex may be unstable under conditions of the assay. The anti-eIF3 serum contains antibodies to eIF4GI [14]. Therefore, the high molecular weight protein detected in the immunoblots probed with this serum likely represents eIF4GI. The presence of this factor in the pull-down reactions was confirmed by probing the blots with an anti-phospho-eIF4GI antibody (Figure 2C). The lower molecular weight products that reacted with the phospho-eIF4GI antibody are interpreted to be degradation products of the full-length protein. Taken together, the data from the pull-down assays and previous results show that the interactions between NV VPg and eIFs and between MNV-1 VPg and eIFs are the same.

### VPg and eIFs co-precipitate from MNV-1 infected RAW 264.7 cells

**Figure 3:**
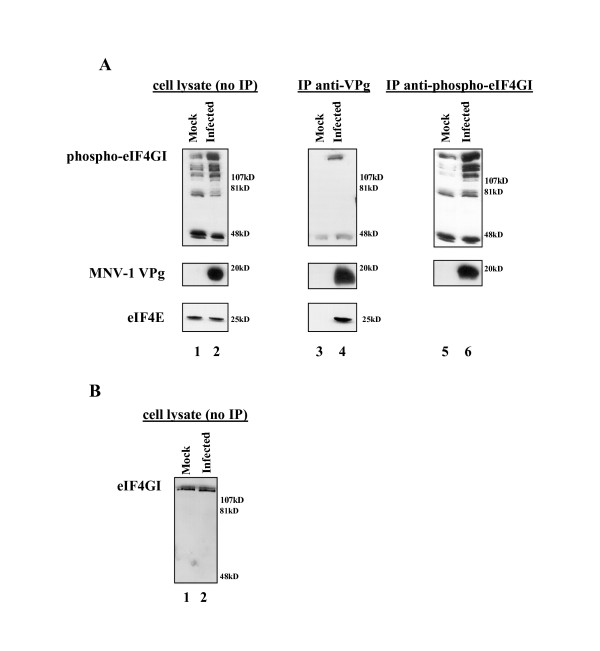
**Phospho-eIF4GI and eIF4E co-immunoprecipitate with VPg from MNV-1 infected RAW 264.7 cells**. A) Mock infected or infected cell lysates were subjected to immunoprecipitation with anti-MNV-1 VPg (lanes 3 and 4) or anti-phospho-eIF4GI (lanes 5 and 6). Immunoblots were probed with the relevant antibody indicated to the left of the panels. Lanes 1 and 2 are control lysates that did not receive antibody. B) Mock infected or infected cell lysates probed with anti-eIF4GI.

To investigate interactions between VPg and eIFs in infected cells, RAW 264.7 cells were infected with MNV-1 and immunoprecipitations (IP) were performed following established protocols [13,15]. MNV-1 VPg was present in infected but not mock infected cell lysate (Figure 3A, lane 2, middle panel) and antibodies to VPg immunoprecipitated VPg (Figure 3A, lane 4, middle panel) and phospho-eIF4GI (Figure 3A, lane 4, top panel). The polar nature of VPg results in a somewhat diffuse migration in SDS polyacrylamide gels instead of a sharp band (Daughenbaugh and Hardy, unpublished observations). In the reciprocal experiment, anti-phospho-eIF4GI antibody precipitated phospho-eIF4GI (Figure 3A, lanes 5 and 6, top panel) and MNV-1 VPg (Figure 3A, lane 6, middle panel). This demonstrates MNV-1 VPg binds eIF4GI in virus-infected cells.

Three additional observations about eIF4GI can be made from these experiments. First, there was a reproducible increase in the amount of phospho-eIF4GI in infected cells compared to mock infected cells (Figure 3A lanes 1, 2, 5 and 6, top panels). However, the levels of total eIF4GI, irrespective of its phosphorylation status, were similar in mock and infected cell lysates (Figure 3B) suggesting that differences in the amount of phospho-eIF4GI cannot be attributed to increased expression of eIF4GI. It has been reported that the amount of eIF4GI phosphorylated at serines 1108, 1148 and 1192 increases in response to serum stimulation, and phosphorylation at these sites is modulated by the N terminal third of the protein [16]. Potential mechanisms were proposed, including de-repression of eIF4GI by direct phosphorylation of the N terminus, or alternatively, interactions with an eIF4GI binding partner to expose the C terminal domain to kinase activity [16]. It is conceivable that MNV-1 infection could result in activation of kinases that modulate the activity of eIF4GI. An alternative and not mutually exclusive possibility is that direct interactions between eIF4GI and VPg alter the conformation of eIF4GI to a form where the C terminal serine residues are phosphorylated.

A second observation is that several eIF4GI degradation products were found in cell lysate (Figure 3A, lanes 1 and 2, top panel), or when protein was immunoprecipitated with anti-phospho-eIF4GI antibody (Figure 3A, lanes 5 and 6, top panel). In contrast, anti-VPg antibody exclusively precipitated full-length eIF4GI. If the interaction between VPg and eIF4GI is direct, these data suggest a binding site for VPg could reside in the N-terminal two-thirds of eIF4GI, since the phospho-eIF4GI antibody recognizes the C terminal third of the protein. If the interaction between VPg and eIF4GI is indirect, full length eIF4GI could have been brought down by a potential direct interaction between VPg and eIF4E because an eIF4E binding site resides in the N terminus of eIF4GI [17].

Finally, the presence of identical degradation products in both mock and infected cell lysates suggests that, in contrast to FCV infections [18], eIF4GI is not detectably cleaved during MNV-1 infection. Although we have not performed extensive analysis of eIF4GI cleavage, these data are consistent with reports that recombinant norovirus 3C protease (MD-145 strain) does not cleave eIF4GI in vitro [19]. This degradation pattern was not observed when mock and infected cell lysates were probed with antibody to total eIF4GI (Figure 3B). The reason for this discrepancy is not entirely clear, but we have observed some variability in the stability of eIF4GI in RAW 264.7 cells lysates. The degradation products also could be C-terminal fragments of the protein not recognized by the eIF4GI antibody made to an N-terminal peptide that was used to detect the full-length protein.

Goodfellow and co-workers recently reported that FCV VPg co-precipitates eIF4E from infected cells, and that this interaction is necessary for translation of FCV VPg-linked RNA in vitro [6]. To determine if MNV-1 VPg was associated with eIF4E in infected cells, we immunoprecipitated VPg from infected cell lysates and probed samples by immunoblot for eIF4E. eIF4E was found in similar quantities in mock and infected cell lysates in the absence of antibody (Figure 3A, lanes 1 and 2, bottom panel), and co-precipitated with VPg from infected cells (Figure 3A, lanes 3 and 4, bottom panel). These data indicate that MNV-1 VPg and eIF4E interact in infected cells. Further studies are necessary to determine if this interaction with MNV-1 VPg is direct, or if eIF4E is detected as a result of interactions with other components of eIF4F.

## Discussion

We and others put forth the idea that VPg functions in translation initiation on VPg-linked RNA through interactions with eIFs [3,5,6,20]. The data presented here provide experimental evidence of interactions between VPg and eIFs in norovirus infected cells, and strengthen the assertion that the interactions previously defined for NV VPg by cell-free assays are relevant and functional.

eIF3, eIF4GI, and eIF4E were detected in GST pull-down eluates of both NV VPg [5] and MNV-1 VPg (this study), and eIF4GI and eIF4E were found in co-immunoprecipitates with VPg from MNV-1 infected cells. While these assays do not prove a direct interaction between VPg and these translation initiation factors, they strongly suggest VPg interacts with one or more components of the eIF4F complex. These observations lend support to previous suggestions that VPg is a protein cap analogue with respect to binding interactions with eIF4F. We further suggest that the role of VPg in ribosome recruitment is more complex than providing a target for eIF4F binding. VPg is a 15 kD protein covalently linked to the 5' end of an RNA that has a 10 nucleotide 5' UTR. If VPg functions simply as a protein cap, then by analogy to capped mRNA, translation initiation at nucleotide 11 would be inefficient [21]. Moreover, direct interactions between VPg and eIF3 [5] point to the potential involvement of VPg in 43S pre-initiation complex recruitment. Sequence conservation between VPg and initiation factor eIF1A, a factor important in AUG codon recognition and ternary complex recruitment [22], has been noted for NV VPg [23], and a similar degree of conservation exists between MNV-1 VPg and eIF1A (data not shown). We have suggested a model of translation initiation on NV VPg-linked RNA that we now extend to MNV-1, whereby a direct interaction between VPg and eIF3, and likely components of eIF4F, positions the ribosome at the initiator AUG without ribosome scanning. This model implies numerous concerted interactions between norovirus VPg and eIFs, including the 40S ribosome, additional to the interactions reported so far.

Studies with animal calicivirus strains in the *Vesivirus *genus of the family, particularly FCV, have contributed a large body of data on mechanisms of calicivirus replication [19,24-26]. These data provide a good foundation on which to build models of norovirus replication by analogy. However, these analogies have limitations, largely due to the genetic divergence between animal caliciviruses and noroviruses. For example, FCV VPg and NV VPg share only 18% amino acid identity, and thus far we and others [6] have been unable to detect interactions between FCV VPg and eIF3 or eIF4GI in GST pull-down assays. We have not performed extensive binding studies with FCV VPg to determine what interactions may occur that are different from those observed with the norovirus VPg, but it is probable that specific interactions and mechanistic details differ between the animal caliciviruses and noroviruses. These observations exemplify the importance of MNV-1 as a molecular model for human noroviruses, as interactions between MNV-1 VPg and eIFs precisely mimic those reported for NV VPg.

## Methods

### RAW 264.7 Cells

RAW 264.7 cells (ATCC TIB-71) were maintained in DMEM containing 10% FBS (Atlanta Biologicals), 10 mM Hepes, 4 mM L-glutamine, 4.5 g/L glucose, and 1.5 g/L sodium bicarbonate.

### Construction of GST-MNV-1 VPg and GST pull-down assays

MNV-1 VPg was amplified from pSPORT-T7-MNV-1 (gift from H. Virgin, Washington University School of Medicine) using the primers MNV-VPg/BamHI(+) 5'-cgcggatccggaaagaagggcaagaac-3' and MNV-VPg/XhoI(-) 5'-ccgctcgagctcaaagttgatcttctcg-3'. Restriction sites for cloning are underlined. Reactions were assembled using the KOD kit (Novagen) according to instructions provided by the manufacturer. Amplification conditions consisted of 25 cycles of 98°C for 15 seconds, 58°C for 3 seconds, and 72°C for 5 seconds. The amplification product was cloned into pGEX-4-T1 (GE Amersham Biosciences) using BamHI and XhoI restriction enzymes. Expression and purification of GST-MNV-VPg, as well as pull-down assays using RAW 264.7 cell lysates, were performed as described previously [5].

### Immunoprecipitations

RAW 264.7 cells were grown to 90% confluence in 10 cm dishes, and infected with MNV-1 for 17 hours at a multiplicity of infection of 1.5 pfu/cell. Cells were harvested in 1 mL of cold medium and pelleted for 5 minutes at 3000 × *g *at 4°C. Pellets were washed twice in cold phosphate-buffered saline, and lysed by incubation on ice for 30 minutes in 500 μL IP buffer containing 20 mM Tris-HCl pH 7.5, 50 mM NaCl, 0.1% NP-40, 10 mM β-glycerophosphate, 5 mM NaF, 2.5% glycerol, and one mini protease inhibitor cocktail tablet (Roche Biochemicals). Lysates were clarified by centrifugation for 5 minutes at 10,000 × *g *at 4°C. Ten μl (2%) of each sample was retained as controls and not subjected to immunoprecipitation. The remaining lysate was incubated with the indicated antibody and rotated gently for two hours at 4°C. Anti-phospho-eIF4GI (Cell Signaling Technologies) was used at a dilution of 1:100 and anti-MNV-1 VPg (provided by Kim Green, NIAID) was used at a dilution of 1:1000. Thirty μl of Gamma Bind Plus Sepharose beads (GE Amersham Biosciences) prepared in IP buffer were added, and complexes were rotated for one hour at 4°C. Beads were collected by centrifugation for 2 minutes at 500 × *g *at 4°C, and then washed four times with wash buffer containing 20 mM Tris-HCl pH 7.5, 50 mM NaCl, and 0.1% NP-40. Complexes were eluted from the beads by incubation on ice for 10 minutes in 25 μl elution buffer (0.75% wt/vol glycine in water, pH 2.2). The beads were collected for 2 minutes at 500 × *g *at 4°C. Supernatants were transferred to fresh tubes containing 0.9 μl 2 M Tris base.

### Western immunoblots

Immunoprecipitation and pull-down samples were subjected to SDS-PAGE and transferred to nitrocellulose, blocked in 10% nonfat dry milk and incubated with the indicated antibody overnight at room temperature. Antibodies used were anti-phospho-eIF4GI at 1:1000, anti-S6 ribosomal protein and anti-eIF4E (both from Cell Signaling Technologies) at 1:1000, anti-eIF3 (kindly provided by John Hershey, UC Davis) at 1:2000, anti-eIF4GI (N-20, Santa Cruz Biotechnology) at 1:1000, and anti-MNV-1 VPg at 1:2000. Appropriate horseradish peroxidase-conjugated secondary antibodies were used at dilutions of 1:3000. Blots were developed with ECL chemiluminescent substrate (GE Amersham Biosciences) and exposed to film for similar lengths of time.

## Competing interests

The author(s) declare that they have no competing interests.
